# Thoracic epidural anesthesia reverses sepsis-induced hepatic hyperperfusion and reduces leukocyte adhesion in septic rats

**DOI:** 10.1186/cc7965

**Published:** 2009-07-13

**Authors:** Hendrik Freise, Fritz Daudel, Christina Grosserichter, Stefan Lauer, Juergen Hinkelmann, Hugo K Van Aken, Andreas W Sielenkaemper, Martin Westphal, Lars G Fischer

**Affiliations:** 1 Department of Anesthesiology and Intensive Care, University Hospital of Muenster, Albert-Schweitzer-Strasse 33, 48149 Muenster, Germany; 2 Department of Intensive Care Medicine, Inselspital, University of Bern, Freiburgstrasse, 3010 Bern, Switzerland; 3 Department of Anesthesiology and Intensive Care Medicine, St. Theresien-Hospital Saarbrücken, Rheinstraße 2, 66113 Saarbrücken, Germany

## Abstract

**Introduction:**

Liver dysfunction is a common feature of severe sepsis and is associated with a poor outcome. Both liver perfusion and hepatic inflammatory response in sepsis might be affected by sympathetic nerve activity. However, the effects of thoracic epidural anesthesia (TEA), which is associated with regional sympathetic block, on septic liver injury are unknown. Therefore, we investigated hepatic microcirculation and inflammatory response during TEA in septic rats.

**Methods:**

Forty-five male Sprague-Dawley-rats were instrumented with thoracic epidural catheters and randomized to receive a sham procedure (Sham), cecal ligation and puncture (CLP) without epidural anesthesia (Sepsis) and CLP with epidural infusion of 15 ul/h bupivacaine 0.5% (Sepsis + TEA). All animals received 2 ml/100 g/h NaCl 0.9%. In 24 (n = 8 in each group) rats, sinusoidal diameter, loss of sinusoidal perfusion and sinusoidal blood flow as well as temporary and permanent leukocyte adhesion to sinusoidal and venolar endothelium were recorded by intravital microscopy after 24 hours. In 21 (n = 7 in each group) separate rats, cardiac output was measured by thermodilution. Blood pressure, heart rate, serum transaminase activity, serum TNF-alpha concentration and histologic signs of tissue injury were recorded.

**Results:**

Whereas cardiac output remained constant in all groups, sinusoidal blood flow increased in the Sepsis group and was normalized in rats subjected to sepsis and TEA. Sepsis-induced sinusoidal vasoconstriction was not ameliorated by TEA. In the Sepsis + TEA group, the increase in temporary venolar leukocyte adherence was blunted. In contrast to this, sinusoidal leukocyte adherence was not ameliorated in the Sepsis + TEA group. Sepsis-related release of TNF-alpha and liver tissue injury were not affected by Sepsis + TEA.

**Conclusions:**

This study demonstrates that TEA reverses sepsis-induced alterations in hepatic perfusion and ameliorates hepatic leukocyte recruitment in sepsis.

## Introduction

The liver is critically involved in a multitude of vital physiological processes and contributes to the host's immune reaction in systemic inflammatory response and sepsis [1-3]. Impaired microcirculation and intrahepatic inflammatory reaction are hallmarks in primary and secondary hepatic injuries [4-6]. In severe sepsis and trauma, liver injury is associated with increased mortality and length of hospital stay [7-10]. The hepatic immune response determines pathogen clearance and the systemic immune reaction [1,5,11]. After prolonged inflammation, hepatic immune dysfunction contributes to mortality [12]. Protection of liver function is therefore crucial to the maintenance of homeostasis in perioperative and critical care medicine.

Sympathetic nerve activity plays a crucial role in hepatic injury and immune response. Increased sympathetic tone alone induces intrahepatic inflammation and liver injury in healthy mice, whereas sympathetic denervation reduced perioperative hepatic injury [13-15]. In sepsis, both α- and β-adrenoreceptors impair hepatic function and immune response [16-18].

Thoracic epidural anesthesia (TEA) promotes postoperative intestinal recovery and reduces cardiovascular mortality, most probably mediated by regional sympathetic block [19-28]. Recently, TEA has also been shown to ameliorate organ injury and improve outcome in sepsis and necrotizing pancreatitis [28-32]. The hepatic effects of TEA in sepsis, however, have never been subject to investigation.

Therefore, we conducted a randomized, blinded experimental study to test the hypothesis that TEA: improves hepatic microvascular perfusion and attenuates leukocyte activation in sepsis; and influences systemic inflammatory response and liver tissue injury induced by cecal ligation and puncture (CLP) in rats.

## Materials and methods

The study was approved by the animal care committee of the District Government of Muenster. Animals received standard chow and were kept in a 12 hour light-dark-cycle. Food was withheld 12 hours prior to surgery. The animals had free access to water.

Male Sprague-Dawley rats (weighing 275 to 300 g; Harlan-Winkelmann, Borchen, Germany) were anesthetized by isofluran in 50% oxygen. Central venous and arterial lines (0.96 mm once daily; Liquidscan, Ueberlingen, Germany) were introduced. Epidural catheters (0.61 mm once daily) were inserted at L3/L4 and advanced to Th6 [26]. All catheters were exteriorized at the neck of the animal and protected by a swivel device. The cecum was ligated below the ileocecal valve to maintain intestinal continuity and then punctured at two locations with an 18-gauge needle. Subsequently anesthesia was terminated and volume resuscitation was performed using 2 mL/100 g/hour isotonic sodium chloride (NaCl) solution intravenously. The animals were housed individually for the following 24 hours. The correct position of the epidural catheter was confirmed by autopsy after completion of the experiment.

After instrumentation, animals were randomly allocated to one of the three groups by closed envelopes: Sham = sham operation, 15 μl/hour NaCl 0.9% epidural; Sepsis = CLP 24 hours, 15 μl/hour NaCl 0.9% epidural; Sepsis + TEA = CLP 24 hours, 15 μl/h bupivacaine 0.5% epidural. The investigators were not aware of the group assignment.

Twenty-four hours after CLP and sham-laparotomy, mean arterial blood pressure was recorded using a standard transducer (PMSET 1 DT, Becton Dickinson, Germany) and a monitor (Siemens Sirecust 404, Siemens, Germany). Heart rate was derived from the arterial pressure curve. For blood gas analyses, 80 μl blood was withdrawn. Motoric block was quantified using an established motor score derived from the Bromage score and adapted to rats [33].

### Intravital microscopy

Twenty-four hours after sepsis induction, 24 animals (n = 8 per group) were then re-anesthetized and tracheotomized [29]. Intravital microscopy of the left liver lobe was performed as follows: median laparotomy was extended by a left subcostal incision and the hepatic ligaments of the left liver lobe were carefully dissected. The animal was placed in a 110° position on its left side onto the microscope (Eclipse 300, Nikon, Düsseldorf, Germany). The left liver lobe was exteriorized and the lower surface was placed on a microscope slide in a tension free position. Intravenously, 2 μmol/kg sodium fluorescein and 0.2 μmol/kg rhodamine 6 G (Sigma, Deisenhofen, Germany) were used for contrast enhancement.

In each experiment, 10 randomly chosen acini and 10 postsinusoidal venoles were recorded for 30 seconds both with sodium-fluorescein and rhodamine contrast enhancement. Offline image analysis was performed by a blinded investigator (CG) using a computer-assisted image analysis system (AnalySIS, OSIS, Muenster, Germany). Hepatic microcirculation was assessed by the periportal sinusoidal diameter of 10 sinusoids per acinus and the loss of sinusoidal perfusion, defined as the number of non-perfused sinusoids divided by all visible sinusoids of the acinus.

The leukocyte adhesion was evaluated separately in sinusoids and postsinusoidal venoles. Temporary adherent, that is, slowly moving or adhering at the sinusoidal wall for less than 20 seconds, and permanently adherent, that is, adherent for more than 20 seconds, leukocytes were counted in each acinus and expressed as cells/μm^2^. Accordingly, temporarily and permanently adherent leukocytes in the venoles were counted as cells/μm^2 ^venolar endothelium.

### Cardiac output and liver injury

In another set of 21 animals, cardiac output was determined applying the thermodilution technique 24 hours after sepsis. In these animals, a thermocouple catheter (IT21, Physitemp, Clifton, NJ, USA) was introduced into the aortic arch via the left carotid artery during instrumentation. For measurement of cardiac output, the area under temperature curve after injection of 0.3 ml cold saline solution (8°C) was recorded (Cardiac Output pod and Powerlab 4/20, ADInstruments, Spechbach, Germany). The results of three measurements were averaged in each animal. To reduce bias of emotional stress the procedure was mimicked every hour for four hours before measurement.

Liver cell injury was assessed 24 hours after induction of sepsis by measuring serum activities of aspartate aminotransferase and alanine aminotransferase. Blood was withdrawn via aortic puncture and plasma enzyme activity was determined by means of standard enzymatic techniques (Ektachem, Kodak, Stuttgart, Germany).

Specimens of the left liver lobe were collected immediately after death and fixed by immersion in 4% formaldehyde solution. Subsequently, they were dehydrated and embedded in paraffin wax to cut sections at a thickness of 5 μm. Slides were stained with H&E and assessed by an experienced pathologist.

### Serum TNF-α

Twenty-four hours after CLP and sham-procedure respectively, serum concentration of TNF-α was measured by a commercially available anti-rat TNF-α ELISA (BD OptEIA, Cat No. 550734, Becton Dickinson, Heidelberg, Germany) according to the manufacturer's instructions and read-out by a fluorometric plate reader (EL808, BioTek, Bad Friedrichshall, Germany).

### Statistics

Sigmastat 3.0 (Systat Software, Richmond, CA, USA) was used for statistical analysis. Normal distribution and equal variance tests were performed. Sepsis-induced and TEA-related effects were evaluated by one-way analysis of variance (ANOVA) with *post-hoc *Student Newman Keuls test or ANOVA on Ranks with *post-hoc *Dunn's test as appropriate. A *P *< 0.05 was defined as the level of significance. Data are presented as mean ± 95% confidence interval or as median (25%/75% percentiles) as appropriate.

## Results

All animals allocated to the sepsis groups showed signs of lethargy, piloerection, and exudation around the eyes and nose 24 hours after induction of sepsis. Peritoneal inflammation and purulent ascites was present when the abdomen was reopened for intravital microscopy.

**Figure 1:**
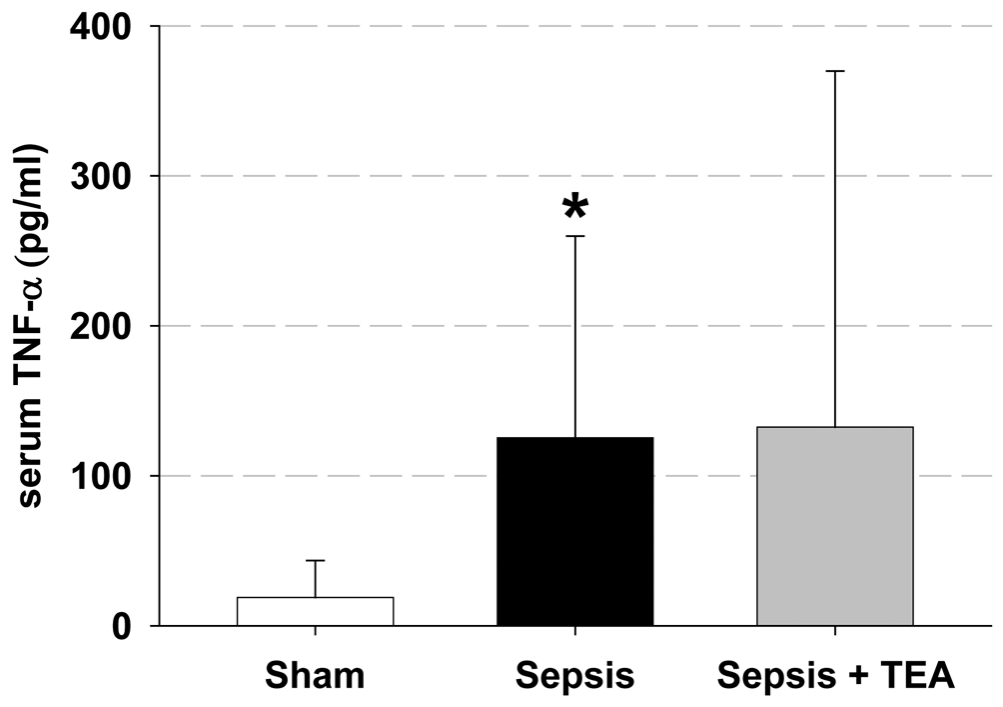
Serum TNF-α. Serum TNF-α 24 hours after induction of sepsis by cecal ligation and puncture and sham procedure respectively. In Sepsis, serum TNF-α was increased compared with Sham (* *P *< 0.05 vs. Sham). Thoracic epidural anesthesia (TEA) did not ameliorate this sign of systemic inflammation. Data (n = 7 in each group) are displayed as mean ± 95% confidence interval.

Compared with the Sham-group, mean arterial blood pressure and heart rate were not affected in the untreated Sepsis or Sepsis + TEA groups. Cardiac output also remained constant both in the Sepsis and Sepsis + TEA groups. Arterial oxygen tension and pH were not altered by sepsis or treatment with TEA (Table 1). Serum-lactate concentrations were increased to 1.25 (1.00/1.50) mmol/l in the Sepsis group compared with 0.8 (0.7/0.9) mmol/l in the Sham group (*P *< 0.05). Leukocyte count dropped from 6430 ± 3099 cells/μL in Sham animals to 2228 ± 1129 cells/μl in the CLP group (*P *< 0.05). In addition, the Sepsis group was characterized by a drop in platelet count compared with the Sham-group (197,000 ± 102,000 cells/μl vs. 338,000 ± 64,000/μl; *P *< 0.05). These parameters were not altered in animals subjected to Sepsis + TEA as compared with the untreated Sepsis group. Serum TNF-α concentration was elevated after 24 hours in the Sepsis-group (*P *< 0.05 vs. Sham). This increase was not ameliorated in the Sepsis+TEA group (Figure 1).

**Table 1:** Cardiorespiratory parameters

	MAP (mmHg)	HR (bpm)	CO (ml/min)	pH	PaO_2_ (mmHg)
Sham	136 ± 10	432 (404/444)	420 ± 74	7.42 ± 0.02	87 ± 10

Sepsis	121 ± 11	468 (447/477)	402 ± 68	7.40 ± 0.09	91 ± 20

Sepsis + TEA	129 ± 16	420 (297/480)	391 ± 152	7.39 ± 0.09	90 ± 11

Twenty-four hours after induction of sepsis by cecal ligation and puncture and sham procedure, respectively. None of these parameters were significantly altered by sepsis or sepsis + thoracic epidural anesthesia (TEA). Data (n = 7) each group are displayed as mean ± 95% confidence interval (CI) or median (25%/75% percentile).

CO = cardiac output; HR = heart rate; MAP = mean arterial pressure; PaO_2 _= partial pressure of arterial oxygen.

### Hepatic microcirculation and leukocyte adherence

**Figure 2:**
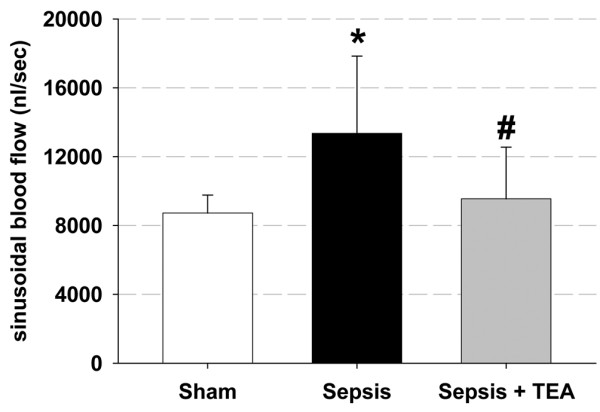
Hepatic microvascular blood flow. Sinusoidal blood flow 24 hour after induction of sepsis by cecal ligation and puncture and sham procedure, respectively. In Sepsis, blood flow was increased compared with Sham (* *P *< 0.05 vs. Sham). Thoracic epidural anesthesia (TEA) reduced blood flow (# *P *< 0.05 vs. Sepsis). Data (n = 8 in each group) are displayed as mean ± 95% confidence interval.

**Figure 3:**
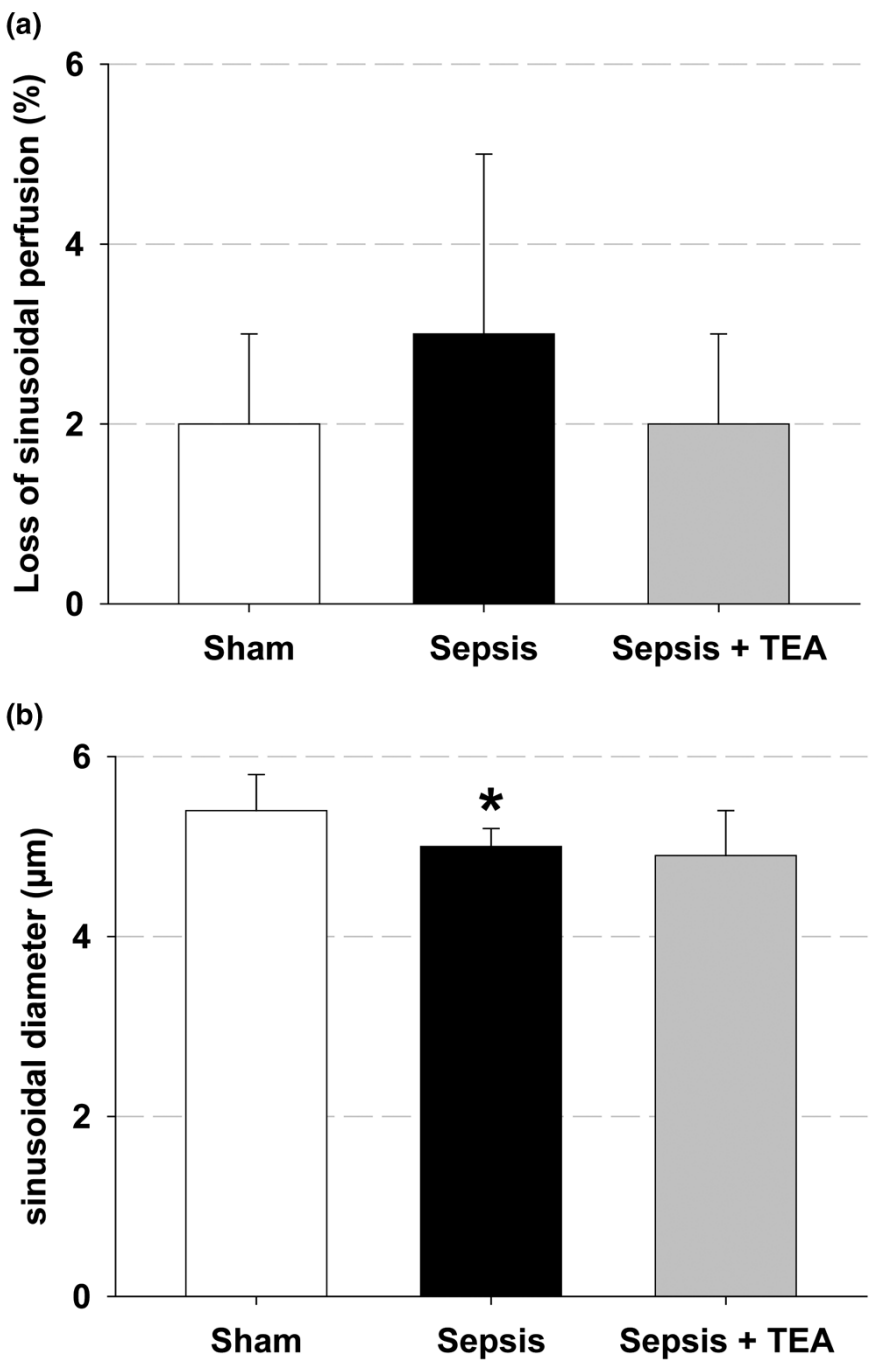
Hepatic microcirculation. **(a) **Percentage of non-perfused sinusoids and **(b) **sinusoidal width 24 hours after induction of sepsis by cecal ligation and puncture and sham procedure, respectively. In Sepsis sinusoidal vasoconstriction occurred (* *P *< 0.05 vs. Sham), which was not influenced in Sepsis + thoracic epidural anesthesia (TEA). Sinusoidal perfusion was neither influenced in Sepsis nor in Sepsis + TEA. Data (n = 8 in each group) are displayed as mean ± 95% confidence interval.

Sinusoidal blood flow increased in the Sepsis group, whereas in the Sepsis + TEA group flow returned to Sham levels (Figure 2). The numbers of perfused sinusoids did not differ between groups. However, in the Sepsis group sinusoidal constriction was induced, which was not influenced in the Sepsis + TEA group (Figure 3).

**Figure 4:**
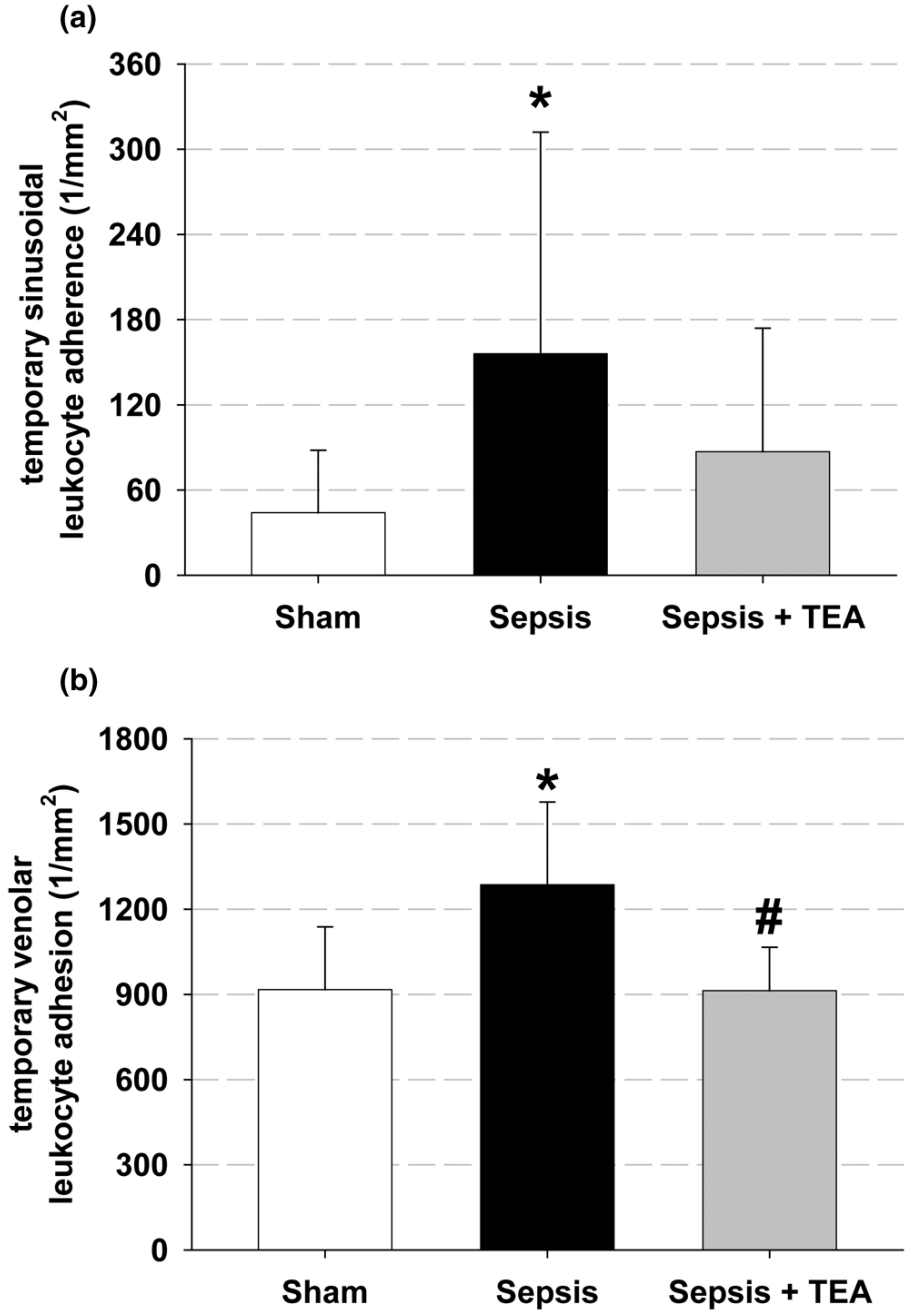
Temporary leukocyte adhesion. Numbers of leukocytes adhering temporarily to the **(a) **sinusoidal and **(b) **postsinusoidal venolar endothelium 24 hours after induction of sepsis by cecal ligation and puncture and sham procedure respectively. In Sepsis, temporary adherence increased both to the sinusoidal and to the venolar endothelium (* *P *< 0.05 vs. Sham). The venolar leukocyte adherence was prevented in Sepsis + TEA (# *P *< 0.05 vs. Sepsis). Data (n = 8 in each group) are displayed as mean ± 95% confidence interval.

Temporary leukocyte adhesion increased in sepsis both in the sinusoids and in the postsinusoidal venules. TEA reduced the temporary venolar leukocyte adhesion significantly, whereas it did not affect the increased sinusoidal adherence (Figure 4). The permanent sinusoidal and venolar leukocyte adherence was neither affected in the Sepsis group, nor in animals subjected to Sepsis + TEA.

### Liver Injury

In the untreated Sepsis group, serum activity of aspartate aminotransferase rose from 275 ± 101 U/l to 454 ± 108 U/l and alanine aminotransferase activity increased from 97 ± 81 U/l to 185 ± 58 U/l (*P *< 0.05 vs. Sham). These increases were not significantly affected by TEA. Similarly, histopathologic examination revealed only mild edema formation and patchy pericentral necrosis in sepsis.

## Discussion

The knowledge about the hepatic effects of TEA is just the beginning. Recent investigation in the pre- and intraoperative period in human and animals revealed conflicting results with respect to hepatic perfusion [34-38]. All these studies were performed in healthy subjects after a single bolus of epidural local anesthetics. The impact of continuous TEA on liver injury in severe sepsis were not investigated.

Hepatic dysfunction in critical illness is still not completely understood. In the current concept of septic liver injury, two phases of dysfunction are distinguished [1]. The early phase is related to hypoperfusion in the presence of hypovolemia and inadequate cardiac output and resolves fast under supportive therapy. The late and persistent dysfunction is characterized by (supra-) normal tissue perfusion.

In this study, the microvascular liver blood flow was significantly increased in the untreated Sepsis group. In the Sepsis + TEA group, sinusoidal blood flow was normalized compared with the untreated Sepsis group. These changes in hepatic perfusion were not correlated to changes in cardiac output, which remained stable both in the Sepsis group and in the Sepsis + TEA group. Furthermore, the effects of TEA on hepatic tissue blood flow were also not associated with altered sinusoidal vasoregulation or increased sinusoidal recruitment.

### Effects of sepsis and TEA on hepatic perfusion

Hepatic macrovascular inflow, although not directly measured in this study, most likely remained constant because cardiac output was not altered. This assumption is supported by numerous studies showing a consistent correlation of cardiac output and macrovascular hepatosplanchnic inflow in sepsis. In human sepsis, macrovascular hepatic inflow rose with cardiac output after therapeutic interventions [39-41]. Both in early and late CLP-sepsis macrovascular hepatic inflow was reduced in parallel with cardiac output [42,43]. Furthermore, in the presence of unchanged or increased cardiac output, hepatic macrovascular inflow also paralleled these changes in cardiac output [42,44,45]. Consequently, the sepsis-induced increase in sinusoidal blood flow and its reversal by TEA are most probably not caused by changes of macrovascular hepatic inflow.

Microvascular tissue perfusion in sepsis, however, is often uncoupled from the systemic circulation. In the clinical therapy of critical illness this dissociation might contribute to the persistent organ failure after hemodynamic stabilization [46]. Earlier studies demonstrated unchanged or even decreased microvascular blood flow in the presence of two to three-fold increased regional blood flow [42,45]. In our study sinusoidal blood flow was increased in the Sepsis group whereas cardiac output was not altered. Intravital microscopy and cardiac output, however, needed to be performed in different sets of animals to minimize interaction between both techniques.

The increase in hepatic microvascular blood flow occurred despite sinusoidal vasoconstriction and consequently was related to increased sinusoidal blood flow velocity. These findings are consistent with an increased arteriolar inflow and are thus the first hint to an impaired hepatic arterial buffer response (HABR) in late polymicrobial sepsis. In HABR, liver arterial blood flow is adapted in response to changes in portal blood flow. Intrahepatic vasodilatation occurs at the level of the preterminal branches of the hepatic artery and is regulated by hydrogen sulfide and adenosine washout by portal blood flow [47,48]. Our results are supported by earlier findings demonstrating impaired HABR and a selective increase in hepatic arterial blood flow in endotoxemia [49,50]. In the Sepsis + TEA group, the sepsis-related increase in liver microvascular blood flow was blunted. It is therefore most likely that the continuous TEA restored HABR. However, this line of interpretation of the presented data is limited by the fact that we did not measure hepatic and portal flow, pressure and resistance separately in this study. Further investigations on the influence of TEA on hepatic blood flow regulation in sepsis and other clinically relevant conditions such as major liver resections are warranted.

In this study, loss of sinusoidal perfusion was not present in the Sepsis group whereas in earlier studies intravital microscopy revealed sinusoidal vasoconstriction and up to 30% reduction in sinusoidal perfusion both in early and late rodent CLP-sepsis [51-53]. Differences in volume resuscitation might partly explain the differing results. In the previous studies an initial bolus of 20 to 60 ml/kg saline solution was administered during 20 to 24 hour CLP-sepsis [53,54]. In our study, volume was infused continuously to a total dose of 144 ml/kg/24 hours. Detection of hypovolemia is often difficult in critical care. In experimental rat sepsis, volume depletion is even harder to exclude. In the present study, mean arterial pressure and heart rate were not significantly affected. Cardiac output remained stable both in untreated rats and in the sepsis + TEA group. The stable systemic hemodynamic parameters combined with stable acid-base-balance and a well-maintained microvascular perfusion in the liver suggests a sufficient resuscitation in this model. This clinically relevant infusion regimen might have prevented loss of perfused sinusoids and contributed to increased tissue blood flow in our study.

### Effects of sepsis and TEA on leukocyte adhesion

In the Sepsis + TEA group, sepsis-induced temporary leukocyte adhesion to the venolar endothelium decreased, whereas temporary sinusoidal leukocyte adhesion was not prevented. In contrast, the permanent leukocyte adhesion after 24 hour of sepsis was neither affected in the Sepsis group nor in the Sepsis + TEA group. This observation is in line with prior findings of time-dependent pattern of leukocyte recruitment in rat CLP-sepsis with increased leukocyte adhesion after seven hours and normalized values after 20 hours [55]. In a recent study hepatic neutrophil recruitment declined after eight hours [56]. In contrast to sinusoidal temporary adhesion, hepatic venolar temporary leukocyte adhesion is initiated by selectins [4,57-59].

Reduced venolar rolling in the Sepsis + TEA group might have been related to immune regulatory consequences of splanchnic sympathetic block. The technique of continuous TEA used in this study induced a sympathetic block including hepatic and intestinal sympathetic nerve roots as demonstrated by thermography [26]. This block can be induced as long as 72 hours after catheter placement. There is some evidence of hepatic sympathetic immune regulation supporting this interpretation. Increased sympathetic activity in acute urinary retention results in hepatic inter-cellular adhesion molecule-1 expression [60]. Increased portal norepinephrine in sepsis trigger hepatic release of TNF-α [17,18], which is in turn a prerequisite for venolar temporary leukocyte adhesion [61,62]. Furthermore, TEA has already been shown to reduce temporary adhesion in mesenteric venoles in hemorrhagic shock [63]. Therefore, the abdominal sympathetic block associated with TEA also might have reduced the venolar temporary leukocyte adhesion in sepsis.

Finally, intestinal injury and portal inflow of inflammatory mediators induce secondary hepatic inflammation [1,64,65]. Consequently, the decreased hepatic leukocyte adhesion may be related to the intestinal protection provided by TEA [29,30,66]. The systemic inflammatory response as measured by systemic release of TNF-alpha was not influenced in the Sepsis + TEA group.

Thoracic epidural infusion of local anesthetics is related to a segmental sensoric block, analgesia, and sympathetic block. Each of these aspects, as well as systemically resorbed bupivacaine might contribute to the observed effects of TEA on hepatic microvascular perfusion and leukocyte adhesion. However, the present study does not allow to distinguish or weight these potential mechanisms or to separate primarily hepatic effects from those secondary to intestinal effects of TEA.

## Conclusions

In this study, TEA ameliorated the sepsis-induced increase in microvascular liver blood flow and attenuated leukocyte recruitment. These results suggest an altered regulation of liver blood flow and a modified intrahepatic immune response during continuous TEA in sepsis. The consequences of TEA with respect to liver injury, remote organ dysfunction and outcome needs to be further explored.

## Key messages

• TEA does not affect cardiac output in late sepsis.

• TEA reverses hepatic hyperperfusion in late sepsis, probably by restoring hepatic arterial buffer response.

• TEA ameliorates intrahepatic temporary leukocyte adhesion in late sepsis.

## Abbreviations

ANOVA: analysis of variance; CLP: cecal ligation and puncture; ELISA: enzyme-linked immunosorbent assay; H&E: hematoxylin and eosin; HABR: hepatic arterial buffer respsonse; NaCl: sodium chloride; TEA: thoracic epidural anesthesia; TNF: tumor necrosis factor.

## Competing interests

The authors declare that they have no competing interests.

## Authors' contributions

HF contributed to design, funding, data acquisition, statistical analysis and drafted the manuscript. FD contributed to the design of the study. CG participated in data acquisition and analysis. SL participated in study planning, data acquisition and statistical analysis. JH contributed to data analysis and to the manuscript. HVA participated in design, funding analysis and manuscript drafting. AWS contributed to funding of the study and participated to planning and statistical analysis. MW contributed to data analysis and drafting of the manuscript. LGF took part in data acquisition and drafting of the manuscript.
